# Editorial: Epigenetic basis of circadian rhythm and metabolism

**DOI:** 10.3389/freae.2025.1736716

**Published:** 2025-11-18

**Authors:** Rasime Kalkan, Peter S. Reinach, Zachary Harvanek, Dongsheng Yan

**Affiliations:** 1 Faculty of Medicine, European University of Lefke, Lefke, Türkiye; 2 Basic Medical College, Wenzhou Medical University, Wenzhou, China; 3 Yale University, New Haven, United States; 4 Wenzhou Medical University, Wenzhou, China

**Keywords:** circadian rhythm, metabolism, hormones, sleep, regulation

Circadian rhythms play an important role in regulating several physiological and metabolic processes. The internal molecular clock plays a pivotal role in maintaining including sleep–wake cycles, hormone secretion, body temperature, and metabolism rhythms. Circadian rhythms are changes in the body, brain, and behavior that occur on a 24-h cycle. Circadian rhythms affect several vital bodily processes, including hormone production, digestion, eating patterns, and body temperature regulation (Takahashi, 2017). The clock machinery has a complex transcriptional–translational feedback loop and clock genes like CLOCK, BMAL1, PER, and CRY, together regulate the rhythmic expression of thousands of downstream genes, many of which participate in metabolic pathways.

Environmental factors, including diet, shift work, and ageing, affect epigenetic reprogramming of the circadian clock. Growing evidence indicates that pharmacological manipulation of chromatin modifiers or sirtuins can partially restore circadian and metabolic balance. Thus, therapeutic interventions against metabolic and age-related diseases might be achieved by targeting the epigenetic-circadian-metabolic network. The goal of this Research Topic is to compile the most recent discoveries and developments in chronobiology and epigenetics.

There is an interaction between epigenetic modifications and circadian rhythms (Aguilar-Arnal and Sassone-Corsi, 2013; Dhaka et al., 2025). Mutations or epigenetic alterations on core circadian clock genes (BMAL1, CLOCK, CRY, PER) can cause alterations in the regulation of the brain, heart, muscle, liver, adipose tissue, pancreas, intestine, and, immune system, glucose homeostasis, lipid metabolism, the risk of hospitalized respiratory infections, and myocardial infarction (Guo et al.; Zhu et al.; Reinke and Asher, 2019; Panda, 2016).

Christopher et al. demonstrated that histone deacetylase 3 (HDAC3) regulates the diurnal rhythms of claudin expression and intestinal permeability. ​ The study showed that HDAC3 is an epigenetic regulator that represses claudin expression by deacetylating histones, thereby influencing diurnal rhythms and intestinal permeability. ​ Claudin genes exhibit diurnal rhythmicity, with expression levels fluctuating across the day-night cycle, impacting intestinal permeability. HDAC3 represses claudin expression and promotes diurnal rhythms by deacetylating histones, particularly H3K9ac and H3K27ac(Christopher et al.). ​ Loss of HDAC3 in IECs increases claudin expression, disrupts diurnal rhythms, and leads to higher intestinal permeability. ​ This study highlights the importance of circadian regulation in maintaining intestinal homeostasis and suggests that targeting HDAC3 could offer therapeutic potential for gastrointestinal disorders linked to circadian disruption (Christopher et al.). ​

Zhu and colleagues showed that sleep behaviours, including sleep duration, insomnia, chronotype, and daytime sleepiness, were also linked to respiratory infection risk, with healthier sleep behaviours associated with lower risks. ​ Chronotype significantly modified the relationship between Hb and respiratory infection risk, with late chronotypes showing a stronger association between abnormal Hb levels and respiratory infection risk. ​ Their study highlights the importance of maintaining balanced Hb levels and healthy sleep behaviors to reduce the risk of respiratory infections. However, it notes limitations such as reliance on self-reported sleep data, single Hb measurements, and the focus on hospitalized cases rather than community infections (Zhu et al.).

In this Research Topic, Gu and colleagues investigated the relationship between Circadian Syndrome (CircS) and Metabolic Syndrome (MetS) with psoriasis, comparing their predictive abilities using data from the NHANES surveys (2005–2006 and 2009–2014). They showed CircS provides a more comprehensive framework for understanding psoriasis risk compared to MetS, emphasizing the importance of circadian rhythm disruptions and lifestyle factors in psoriasis development. ​ The study highlights the need to monitor CircS components—particularly elevated blood pressure, depressive symptoms, and elevated waist circumference—to better manage psoriasis risk (Gu et al.).

This Research Topic brings together studies investigating the epigenetic regulation of circadian rhythms and metabolism in a variety of biological systems. We seek to discuss new mechanisms, molecular interactions, and translational insights that continue to advance our understanding of how the epigenome regulates temporal and metabolic physiology. Together, these contributions will advance the time-metabolism relationship and open new avenues for chronotherapy and epigenetic-based interventions in metabolic disease. This Research Topic is intended to provide context and provoke further work in the field of epigenetics and circadian rhythm.
